# Emerging and Re-Emerging Zoonoses of Dogs and Cats

**DOI:** 10.3390/ani4030434

**Published:** 2014-07-15

**Authors:** Bruno B. Chomel

**Affiliations:** Department of Population Health and Reproduction, School of Veterinary Medicine, University of California, Davis, CA 95616, USA; E-Mail: bbchomel@ucdavis.edu; Tel.: +1-530-752-8112

**Keywords:** zoonoses, dog, cat, emerging diseases

## Abstract

**Simple Summary:**

Dogs and cats have been sharing our environment for a long time and as pets they bring major psychological well-being to our modern urbanized society. However, they still can be a source of human infection by various pathogens, including viruses, bacteria, parasites, and fungi.

**Abstract:**

Since the middle of the 20th century, pets are more frequently considered as “family members” within households. However, cats and dogs still can be a source of human infection by various zoonotic pathogens. Among emerging or re-emerging zoonoses, viral diseases, such as rabies (mainly from dog pet trade or travel abroad), but also feline cowpox and newly recognized noroviruses or rotaviruses or influenza viruses can sicken our pets and be transmitted to humans. Bacterial zoonoses include bacteria transmitted by bites or scratches, such as pasteurellosis or cat scratch disease, leading to severe clinical manifestations in people because of their age or immune status and also because of our closeness, not to say intimacy, with our pets. Cutaneous contamination with methicillin-resistant *Staphylococcus aureus*, *Leptospira* spp., and/or aerosolization of bacteria causing tuberculosis or kennel cough are also emerging/re-emerging pathogens that can be transmitted by our pets, as well as gastro-intestinal pathogens such as *Salmonella* or *Campylobacter*. Parasitic and fungal pathogens, such as echinococcosis, leishmaniasis, onchocercosis, or sporotrichosis, are also re-emerging or emerging pet related zoonoses. Common sense and good personal and pet hygiene are the key elements to prevent such a risk of zoonotic infection.

## 1. Introduction

Dogs and cats have been human companions for more than 10,000 years. They have been sharing our environment and have gained a major status as “pets” in our modern, very urbanized society. Since the middle of the 20th century, they are more and more considered as “family members” within households; not to mention sometimes as substitutes for children. However, cats and dogs still can be a source of human infection by various pathogens, including viruses, bacteria, parasites, and fungi. The present manuscript analyzes and reviews the zoonotic pathogens that have recently emerged (or re-emerged) from our companion animals. This review excludes the impact of exotic and pocket pets, which deserve a distinct review.

Pet dog and cat populations have substantially increased in the developed world and it is estimated that dogs and cats are present in more than 50% of households in the USA (2012 estimated dog population: 71 million, 2012 estimated cat population: 74 million [1] and Europe (2012 estimated dog population: 75 million, 2012 estimated cat population: 90 million; with 70 million households owning a pet [2]. A similar trend is emerging in Asia in countries such as Japan, Taiwan and even China, as the Chinese population owning dogs and cats increased from 5% and 14%, respectively, in 1999 to an estimated 7% and 15% in 2004, respectively [3].

## 2. Viral Zoonoses

### 2.1. Rabies

Despite the fact that canine rabies has come under control in many parts of the developed world, it is still a major problem with pet dogs and to a lesser extent pet cats in many other parts of the world. More than 99% of human cases of rabies are still related to dog exposure [4]. An example of such an emergence or re-emergence is illustrated by the introduction of rabies in the Island of Bali, Indonesia, in 2008, leading to more than 130 human deaths [5]. Similarly, countries that have eradicated dog rabies are not exempt of accidental re-introduction by pet trade or adoption of dogs (often puppies) in rabies endemic countries and brought back home [6]. Such cases have been reported in countries such as France [7] or the USA [8]. Rabies vaccination of dogs is still the key control measure to prevent the dispersal of rabies.

### 2.2. Cowpox

Cowpox is an old disease that gained fame when it was shown by Jenner that it was protecting young farmers from smallpox and led to the first human vaccine. Rather uncommon now in cattle in developed countries, it has been demonstrated that in fact rodents are the natural reservoir of the virus, cows being victims of the viral infection, just like humans [9]. However, more human cases are now related to cat exposure in the United Kingdom or Western Europe. For domestic cats, more than 400 cases of cowpox infections have been described [10]. Both animals and humans reveal local exanthema on arms and legs or on the face. Although cowpox is generally regarded as a self-limiting disease, immunosuppressed patients can develop a lethal systemic disease resembling smallpox. In domestic cats, multiple skin lesions (primarily seen on head, oral cavity, neck, forelimb, or paws), conjunctivitis, or purulent ocular discharge develop upon infection. Systemic infections may occur if inner organs as the lungs (necrotizing pneumonia), co-infections or immune-deficiency are involved [10]. It is estimated that more than 50% of human cases of cowpox in the United Kingdom are related to exposure to cats [11].

### 2.3. Avian Influenza

Cases of influenza H5N1 have been reported in domestic cats in Europe (Austria, Germany) and several countries in Asia [12]. An outbreak was also reported in captive tigers in a Zoological garden in Thailand. These tigers had been fed with chickens that died of avian influenza H5N1 [12]. A few cases of cats infected with H1N1 in the USA [13] and Italy [14] have also been reported. An outbreak of avian influenza H3N2 was reported in pet dogs in South Korea in 2007. The epidemic of H3N8 in greyhounds in the USA is related to an equine influenza virus and has not been shown to be zoonotic. The role of cats and dogs as a source of human infection seems limited. However, cats were fully susceptible to experimental infection and infected cats were able to infect naive cats. The role of dogs seem even more limited for the dispersal of avian influenza to humans. Rather, humans may be the source of pet infection, as suggested for H1N1 infection [13].

### 2.4. Noroviruses, Rotaviruses

There are only anecdotal links exist between dog gastrointestinal viruses and human infections. Human norovirus (NoV) sequences were recently detected in fecal samples from pet dogs that had been in direct contact with humans with NoV gastroenteritis, suggesting that human NoVs can at least survive in the gastrointestinal tract of dogs [15]. However, canine noroviruses (CaNoV) have also been recently identified [16] and a few reports have suggested the role of dogs in the dispersal of noroviruses. CaNoV may infect humans, and small animal veterinarians are at an increased risk, as antibodies to CaNoV were found in 22.3% of 373 small animal veterinarians and 5.8% of 120 age-matched control individuals [17]. A report of infection by an animal-like strain of rotavirus (PA260/97) was diagnosed in a child with gastroenteritis in Palermo, Italy, in 1997. Sequence analysis of VP7, VP4, VP6, and NSP4 genes showed resemblance to a G3P [6] canine strain identified in Italy in 1996 [18]. Similarly, a strain similar to a rare canine strain G3P [6] was also detected from a one year old child in Brazil [19].

## 3. Bacterial Zoonoses

### 3.1. Bite-Transmitted Bacterial Infections

*Pasteurella multocida*, *Capnocytophaga canimorsus* are among the emerging or re-emerging pathogens that cause severe diseases in high-risk groups: immunocompromised individuals, elderly people, organ transplant recipients, or cancer patients [20,21]. New modes of infection have been identified in recent years either by kissing pets, giving palliative care, or sleeping with the pets [22,23,24]. For *C.* *canimorsus*, the bacterial isolates are classifiable into two main groups (I and II) with differing γ-glutamyl aminopeptidase activity [25]. Strains from human patients belonged unevenly to group I, possibility suggesting that group I can be transmitted to humans, and group II is indigenous only to the oral cavities of dogs and cats [25].

### 3.2. Cat Scratch Disease

*Bartonella henselae* has been identified only in the early 1990s as the etiological agent of cat scratch disease (CSD), causing fever and enlarged lymph nodes in humans [26]. In less than 15% of the CSD cases, complicated forms, including visceral lesions, encephalitis and endocarditis have been reported. In immunocompromised individuals, it causes bacillary angiomatosis characterized by cutaneous vasculo-proliferative lesions that can be fatal if not properly treated with antibiotics (doxycycline, gentamycine, erythromycin) for several weeks to three months [27]. Cat fleas are the main vector and transmit infection through flea feces containing infective bacteria, which can survive several days in the environment. Cats transmit the infection to humans through scratches when infective flea feces contaminate their claws. Stray cats and young cats are more likely to be bacteremic and being a source of human infection [26]. The role of dogs as source of human infection is not yet clearly understood, as dogs can be infected by a wide range of *Bartonella* species, besides *B. henselae*.

### 3.3. Methicillin Resistant *Staphylococcus Aureus* (MRSA) Infection

*Staphylococcus aureus* is a common human commensal organism; acquisition of genes encoding an altered penicillin-binding protein confers resistance to beta-lactam antimicrobial drugs. Methicillin-resistant *S. aureus* (MRSA) is often resistant to non-beta-lactam antimicrobial drugs as well. Originally described as an important cause of nosocomial infection, MRSA colonization and infection are now often identified in humans outside healthcare settings [28]. Dogs and cats are more likely to be colonized/infected with *Staphylococcus pseudointermedius* than *S. aureus*, but this pathogen can acquire genes encoding methicillin resistance (*i.e.*, MRSP). Diagnosis of MRSA or MRSP has implications not only for treatment of infected animals, but also for potential zoonotic transmission. There are increasing reports that suggest pet animals may play a role in household MRSA transmission [29,30].

In the United Kingdom, the genomes of 46 multilocus sequence type (ST) 22 MRSA isolates from cats and dogs were sequenced and compared to an extensive population framework of human isolates from the same lineage [31]. Phylogenomic analyses showed that all companion animal isolates were interspersed throughout the MRSA-15 (EMRSA-15) pandemic clade and clustered with human isolates from the United Kingdom, with human isolates basal to those from companion animals, suggesting a human source for isolates infecting companion animals.

A recent study evaluated the prevalence and risk factors for MRSA carriage by pets residing in households with an MRSA-infected person [32]. Ninety-nine pets (47 dogs and 52 cats) from 66 households in which an MRSA-infected patient resided, were screened using a swab protocol and isolates from pets and humans were genotyped using two techniques and compared for concordance. Eleven (11.5%) pets representing 9 (13.6%) households were MRSA-positive, but in only six of these households were the human and animal-source strains genetically concordant. Human infection by strain USA 100 was significantly associated with pet carriage (OR = 11.4 (95% CI 1.7, 76.9); *p* = 0.013). However, the source of MRSA to the pet cannot always be attributed to the human patient. Moreover, the rapid attrition of the odds of obtaining a positive culture from pets over time suggests that MRSA carriage may be fleeting. In conclusion, as stated by Bramble *et al.* [33], “Future work to more clearly elucidate the role of pet animals should be emphasized with resultant endeavors to test interventions to curb the transmission of MRSA between humans and pet animals”.

### 3.4. Leptospirosis

Leptospirosis is a common zoonotic disease affecting dogs and humans. However, it has re-emerged in several parts of the world, associated with both climatic changes and changes in serovars implicated in canine cases. Canine leptospirosis has been described as having re-emerged in North America around the mid-1990s, with a change in the epidemiology of the infecting serovars responsible for the disease emergence [34]. In a retrospective study of 1406 leptospirosis cases in dogs from Ontario, Canada, for the period 1998 to 2006, Alton *et al.* [34] showed a distinctive seasonal pattern of leptospirosis, with more cases occurring during the summer and fall. The temporal trend analysis was consistent with an increasing proportion or re-emergence of seropositive cases of canine leptospirosis since 1998, suggesting that the putative increase in canine leptospirosis has been genuine. Similarly, the Veterinary Medical Database of hospitals showed that the prevalence of leptospirosis in dogs in the USA and Canada, after a marked decrease in the 1970s and low levels in the 1980s, began increasing in the 1990s [35]. Hospital prevalence significantly increased in dogs between 2 and 9.9 years of age (*p* < 0.05) and in male dogs (*p* < 0.05) in each decade since the 1980s. Among weight groups in the most recent decade (2000–2009), dogs weighing <15 pounds had the greatest odds of being diagnosed with leptospirosis (*p* = 0.003). One of the major issues with canine leptospirosis, is that most of the dogs with the disease had sera reacting to serogroups other than icterohaemorrhagiae and canicola, which are contained in the vaccines, as reported in southern Germany [36] or in the USA [37]. In California, infection with *L. pomona* and *L. bratislava* was recognized as a cause of leptospirosis in dogs, which resulted in development of acute renal failure with various degrees of azotemia. Thus, available vaccines in the USA or in Europe did not protect against the most common Leptospira organisms associated with clinical disease. The recent introduction of tetravalent vaccine should help reduce such a risk.

The recommended treatment for optimal clearance of the organism from renal tubules is doxycycline, 5 mg/kg p.o. q12h, for 14 days. Annual vaccination can prevent leptospirosis caused by serovars included in the vaccine and is recommended for dogs at risk of infection.

### 3.5. Tuberculosis (*Mycobacterium bovis*, *M. tuberculosis* and *M. microti*)

The re-emergence in the United Kingdom, Northern Ireland, and in New Zealand of *Mycobacterium bovis*, the causative agent of bovine tuberculosis (bTB), has led to infection of a broad range of mammalian species in addition to domestic cattle, badgers and Australian brushtail possums (*Trichosurus vulpecula*) [38,39]. Since specific tuberculosis legislation was introduced in 2006 in Great Britain, the organism has been increasingly identified in domestic species other than cattle. Diagnostic submissions for mycobacterial culture between 2004 and 2010 showed infection in several mammals, including 116 cats, and seven dogs [39]. Gunn-Moore *et al.* [40] reviewed mycobacterial submissions to AHVLA between 2005–2008 and found *M. microti* in 19% of reported cases, with *M. bovis* in 15%. In Great Britain, isolations of *M. bovis*, *M. microti*, and *M. avium* in cats appear to have discrete, almost entirely non-overlapping geographical distributions, with *M. bovis* isolations concentrated in areas where the bTB is endemic in cattle. Cats commonly presented with single or multiple cutaneous lesions (74%), which were sometimes ulcerated or discharging, located most frequently on the head (54%). Lymph nodes were usually involved (47%), typically the submandibular nodes. Systemic or pulmonary signs were rarely seen (10%–16%). Dog tuberculosis is now less frequently reported. However, transmission of *M. tuberculosis* from infected humans to dogs may still occur, as reported in a few cases in the USA and Europe [41,42,43].

### 3.6. Kennel Cough (Bordetella bronchiseptica)

Several human cases of *Bordetella bronchiseptica* infections have been reported in immunocompromised individuals, especially organ transplant recipients and cancer patients who had been exposed either to dogs or cats that can be healthy carriers of this emerging zoonotic pathogen [44]. Symptoms ranged from asymptomatic carriage to severe pneumonia [45]. Although *B.* *bronchiseptica* infection remains a rare clinical condition among humans, it should be considered as potentially pathogenic when found in airways of immunocompromised patients.

### 3.7. Gastro-Intestinal Pathogens: Salmonella and Campylobacter

Recent studies have shown that dogs fed with raw meat or pig-ear treats were more likely to shed *Salmonella* and *Campylobacter* in the environment and be a source of human contamination. Lenz *et al.* [46] reported that *Campylobacter jejuni* was isolated from 1/42 (2.6%) raw meat-fed dogs. *Salmonella* *enterica* was isolated from 2/40 (5%) of the raw meat feeds, 6/42 (14%) of the raw meat-fed dog feces and none of the dogs that did not receive raw meat (*p* = 0.001). Similarly, nosocomial outbreaks have been reported in veterinary clinics in North America. In 1999 and 2000, three state health departments reported four outbreaks of gastrointestinal illness due to *Salmonella* *enterica* serotype Typhimurium in employees, clients, and client animals from three companion animal veterinary clinics and one animal shelter [47]. More than 45 persons and companion animals became ill. Four independent investigations resulted in the testing of 19 human samples and >200 animal samples; 18 persons and 36 animals were culture-positive for *S. typhimurium*. One outbreak was due to multidrug-resistant *S. typhimurium* R-type ACKSSuT, while the other three were due to multidrug-resistant *S. typhimurium* R-type ACSSuT DT104.

### 3.8. Vector-Borne Zoonoses

Despite the recognition of a wide number of vector borne diseases that can infect pets, such as Lyme disease, ehrlichiosis, or tick borne encephalitis, the risk from pets is not so much from direct transmission of these pathogens to humans as from than their role as carriers of these vectors in the shared environment. For this main reason, these zoonotic pathogens are not presented in detail in this review. Two examples are the role of dogs and cats as source of plague caused by *Yersinia pestis* after sleeping with flea infested pets [22] or the presence of dogs and brown dog ticks (*Rhipicephalus sanguineus*) in playgrounds (under the houses) leading to several cases of Rocky mountain spotted fever cases in Arizona [48].

## 4. Parasitic and Fungal Zoonoses

### 4.1. Echinococcosis

The life cycle of *Echinococcus multilocularis* mainly involves foxes and their rodent prey in ecosystems generally separate from humans [49]. However, as fox and coyote populations have increasingly encroached upon suburban and urban areas, domestic dogs or cats may also become infected when they eat infected wild rodents. *E. multilocularis* is endemic in parts of Europe, much of Russia, the Central Asian republics, and Western China on the old continent and the northwestern portion of Canada, and Western Alaska in the New World. In rural regions of Central North America, the cycle involves foxes and rodents of the genera *Peromyscus* and *Microtus*. The role of foxes in the zoonotic transmission of alveolar echinococcosis appears to be important, as demonstrated by increases in the incidence of human alveolar echinococcosis following the increase in population of foxes in certain parts of Europe.

The annual incidence in humans in endemic areas of Europe has increased from a mean of 0.10 per 100,000 during 1993–2000 to a mean of 0.26 per 100,000 during 2001–2005. There is evidence of parasites spreading from endemic to previously non-endemic areas in North America and North Island, Hokkaido, Japan, due principally to the movement or relocation of foxes and in Europe due to an increase in the fox population densities [50]. The infection of humans by the larval *E. multilocularis* is often the result of association with dogs that have eaten infected rodents. However, domestic cats may also be a potential source of human infection, as shown in Europe. In Germany, a case–control study demonstrated a higher risk of alveolar echinococcosis among individuals who owned dogs that killed game, dogs that roamed outdoors unattended, individuals who were farmers, and individuals who owned cats [51]. In Austria, a study to identify the risk of pet ownership (*i.e.*, cats and dogs) for alveolar echinococcosis investigated the habits and activities of 21 confirmed patients during the period 1967–1997 which were compared with the habits and activities of 84 controls matched by sex, age, and residence [52]. Cat ownership (odds ratio (OR) = 6.47, 95% confidence interval (CI) 1.54–27.29) and hunting (OR = 7.83, 95% CI 1.16–52.77) were independent risk factors associated with alveolar hydatid disease. In France, three (3.7%) *E. multilocularis* infested cats were detected out of 81 necropsied [53]. However, the role of cats in the transmission of *E. multilocularis* may not be as significant as once believed, as studies have shown they are much less susceptible to infection with the parasite than canids [49].

### 4.2. Onchocercosis

Cases of canine ocular onchocercosis, caused by *Onchocerca lupi*, have been reported worldwide, particularly in the United States and Europe. Its zoonotic role has been hypothesized on the basis of the reexamination of two cases of human ocular onchocerciasis and was confirmed in the sub-conjunctival region of the human eye in a patient from Turkey [54]. In the USA, a 22-month-old girl presented with neck pain and stiffness and magnetic resonance imaging showed an extradural mass extending from C2 through the C4 level with moderate to severe compression of the cord [55]. A left unilateral C2–C4 laminectomy was performed revealing an extradural rubbery tumor; a small biopsy was obtained. Examination of stained tissue revealed the presence of a parasitic worm that was identified as a gravid *O. lupi* female; the first report of zoonotic *O. lupi* in the United States. The parasite has been reported in dogs and cats in the western United States, and from people in four cases reported from Europe.

### 4.3. Leishmaniasis

In North America, cutaneous leishmaniasis is endemic within south-central Texas and appears to be spreading northward into the Dallas-Fort Worth metro area, affecting humans, cats, and dogs [56]. Multiple vectors and rodent reservoir hosts exist within Texas, leading to consideration of vector-borne sand fly–based transmission as the primary means of disease spread in this area. More recently, canine visceral leishmaniasis was identified as endemic in the US foxhound population, mainly transmitted through vertical and horizontal (bites) routes in this population, although *Lutzomyia* species in the United States may be involved in transmission. Further study is necessary to determine the likelihood of vector-borne transmission of both cutaneous and visceral leishmaniasis in the United States. Diagnosis is based on qPCR to detect infection and serology to indicate the onset or presence of an antibody-based immune response to *Leishmania* spp. Treatment options include allopurinol, glucantime, and newer, less toxic formulations of amphotericin B, but none of these drugs lead to lifelong sterile cure, and recrudescence of both visceral and cutaneous disease is possible, although more common after infection with *L. infantum* (visceral) or *L. brasiliensis* (cutaneous). Evidence indicates that this disease may be further emerging because of changes in the environment and closer contact between pets and sylvatic ecosystems.

### 4.4. Sporotrichosis

In the United States, sporotrichosis is considered to be endemic to the Mississippi and Missouri River valleys, whereas cases have been reported infrequently in California and the southwestern United States. Zoonotic transmission has been reported from a variety of domestic and wild animals, but is most commonly associated with felines [57]. A major epidemic occurred in Rio de Janeiro, Brazil in the early 2000 where 83.4% of more than 750 people affected reported contact with cats with sporotrichosis, 56% of whom reported a cat scratch or bite [57]. During that epidemic more than 1500 feline cases were diagnosed. Outdoor cats are at highest risk for contracting sporotrichosis. Like humans, cats acquire the disease via penetrating injury by foreign body or by fighting other cats. Veterinarians and cat owners are at increased risk of contracting the disease because of the high level of transmissibility from felines to humans as compared with other animal species. The high level of contagiousness of cats with sporotrichosis is thought to arise from the typically high numbers of organisms present in the lesions. Once the organism gains entry, the typical incubation period is one week to two months in humans, with most cases manifesting within the first three weeks of exposure. The clinical forms of sporotrichosis are categorized as localized (or fixed) cutaneous, lymphocutaneous, disseminated (systemic), and pulmonary. The lymphocutaneous form is the most common clinical presentation, with a primary ulcerative lesion and subcutaneous nodules extending in a linear pattern along the lymphatic channels (sporotrichoid distribution). In cats, sporotrichosis varies from subclinical infection to severe systemic disease with hematogenous dissemination of *Sporothrix schenckii* [58]. The definitive diagnosis of sporotrichosis in both humans and cats requires recovery of the organism from culture. The drug of choice to treat these patients has been oral itraconazole.

## 5. Conclusions

Pet ownership brings major well-being support and the risk of zoonoses is limited when good animal care and appropriate preventive measures are applied in the human environment. However, the risks are not null and some behaviors (kissing, sleeping, being licked, or sharing food or kitchen utensils) or exposure of high-risk group persons may lead to disease carried by companion animals. Better diagnostic tools have also increased our knowledge of the zoonotic or potentially zoonotic pathogens present in our direct environment. Finally, pets represent excellent sentinels to identify the pathogens that can infect humans where they live or when people travel with them.

## References

[1] U.S. Pet Ownership Statistics. https://www.avma.org/KB/Resources/Statistics/Pages/Market-research-statistics-US-pet-ownership.aspx#companion.

[2] Facts and Figures Statistics Underline the Importance of Pet Animals in Society. http://www.fediaf.org/facts-figures/.

[3] China: Changing Attitudes to Pet Ownership Drive Pet Food Sales. http://www.marketresearchworld.net/content/view/281/77.

[4] Fooks A.R., Banyard A.C., Horton D.L., Johnson N., McElhinney L.M., Jackson A.C. (2014). Current status of rabies and prospects for elimination. Lancet.

[5] Townsend S.E., Sumantra I.P., Brum E., Cleaveland S., Crafter S., Dewi A.P., Dharma D.M., Dushoff J., Girardi J., Gunata I.K. (2013). Designing programs for eliminating canine rabies from islands: Bali, Indonesia as a case study. PLoS Negl. Trop. Dis..

[6] Weng H.Y., Wu P.I., Yang P.C., Tsai Y.L., Chang C.C. (2010). A quantitative risk assessment model to evaluate effective border control measures for rabies prevention. Vet. Res..

[7] Gautret P., Ribadeau-Dumas F., Parola P., Brouqui P., Bourhy H. (2011). Risk for rabies importation from North Africa. Emerg. Infect. Dis..

[8] McQuiston J.H., Wilson T., Harris S., Bacon R.M., Shapiro S., Trevino I., Sinclair J., Galland G., Marano N. (2008). Importation of dogs into the United States: Risks from rabies and other zoonotic diseases. Zoonoses Public Health.

[9] Vorou R.M., Papavassiliou V.G., Pierroutsakos I.N. (2008). Cowpox virus infection: An emerging health threat. Curr. Opin. Infect. Dis..

[10] Essbauer S., Pfeffer M., Meyer H. (2010). Zoonotic poxviruses. Vet. Microbiol..

[11] Lawn R. (2010). Risk of cowpox to small animal practitioners. Vet. Rec..

[12] Harder T.C., Vahlenkamp T.W. (2010). Influenza virus infections in dogs and cats. Vet. Immunol. Immunopathol..

[13] Sponseller B.A., Strait E., Jergens A., Trujillo J., Harmon K., Koster L., Jenkins-Moore M., Killian M., Swenson S., Bender H. (2010). Influenza A pandemic (H1N1) 2009 virus infection in domestic cat. Emerg. Infect. Dis..

[14] Fiorentini L., Taddei R., Moreno A., Gelmetti D., Barbieri I., de Marco M.A., Tosi G., Cordioli P., Massi P. (2011). Influenza A pandemic (H1N1) 2009 virus outbreak in a cat colony in Italy. Zoonoses Public Health.

[15] Summa M., von Bonsdorff C.H., Maunula L. (2012). Pet dogs—A transmission route for human noroviruses?. J. Clin. Virol..

[16] Martella V., Lorusso E., Decaro N., Elia G., Radogna A., D’Abramo M., Desario C., Cavalli A., Corrente M., Camero M. (2008). Detection and molecular characterization of a canine norovirus. Emerg. Infect. Dis..

[17] Mesquita J.R., Costantini V.P., Cannon J.L., Lin S.C., Nascimento M.S., Vinjé J. (2013). Presence of antibodies against genogroup VI norovirus in humans. Virol. J..

[18] De Grazia S., Martella V., Giammanco G.M., Gòmara M.I., Ramirez S., Cascio A., Colomba C., Arista S. (2007). Canine-origin G3P[3] rotavirus strain in child with acute gastroenteritis. Emerg. Infect. Dis..

[19] Luchs A., Cilli A., Morillo S.G., Carmona Rde C., Timenetsky Mdo C. (2012). Rare G3P[3] rotavirus strain detected in Brazil: Possible human-canine interspecies transmission. J. Clin. Virol..

[20] Gaastra W., Lipman L.J. (2010). *Capnocytophaga canimorsus*. Vet. Microbiol..

[21] Wilson B.A., Ho M. (2013). *Pasteurella multocida*: From zoonosis to cellular microbiology. Clin. Microbiol. Rev..

[22] Chomel B.B., Sun B. (2011). Zoonoses in the bedroom. Emerg. Infect. Dis..

[23] Kawashima S., Matsukawa N., Ueki Y., Hattori M., Ojika K. (2010). *Pasteurella multocida* meningitis caused by kissing animals: A case report and review of the literature. J. Neurol..

[24] Myers E.M., Ward S.L., Myers J.P. (2012). Life-threatening respiratory pasteurellosis associated with palliative pet care. Clin. Infect. Dis..

[25] Umeda K., Hatakeyama R., Abe T., Takakura K., Wada T., Ogasawara J., Sanada S., Hase A. (2014). Distribution of *Capnocytophaga canimorsus* in dogs and cats with genetic characterization of isolates. Vet. Microbiol..

[26] Chomel B.B., Boulouis H.J., Maruyama S., Breitschwerdt E.B. (2006). *Bartonella* spp. in pets and effect on human health. Emerg. Infect. Dis..

[27] Rolain J.M., Brouqui P., Koehler J.E., Maguina C., Dolan M.J., Raoult D. (2004). Recommendations for treatment of human infections caused by *Bartonella* species. Antimicrob. Agents Chemother..

[28] Cohn L.A., Middleton J.R. (2010). A veterinary perspective on methicillin-resistant staphylococci. J. Vet. Emerg. Crit. Care (San Antonio).

[29] Faires M.C., Tater K.C., Weese J.S. (2009). An investigation of methicillin-resistant *Staphylococcus aureus* colonization in people and pets in the same household with an infected person or infected pet. J. Am. Vet. Med. Assoc..

[30] Weese J.S., Dick H., Willey B.M., McGeer A., Kreiswirth B.N., Innis B., Low D.E. (2006). Suspected transmission of methicillin-resistant *Staphylococcus aureus* between domestic pets and humans in veterinary clinics and in the household. Vet. Microbiol..

[31] Harrison E.M., Weinert L.A., Holden M.T.G., Welch J.J., Wilson K., Morgan F.J.E., Harris S.R., Loeffler A., Boag A.K., Peacock S.J. (2014). A shared population of epidemic methicillin-resistant *Staphylococcus aureus* 15 circulates in humans and companion animals. mBio.

[32] Morris D.O., Lautenbach E., Zaoutis T., Leckerman K., Edelstein P.H., Rankin S.C. (2012). Potential for pet animals to harbour methicillin-resistant *Staphylococcus aureus* when residing with human MRSA patients. Zoonoses Public Health.

[33] Bramble M., Morris D., Tolomeo P., Lautenbach E. (2011). Potential role of pet animals in household transmission of methicillin-resistant *Staphylococcus aureus*: A narrative review. Vector Borne Zoonotic Dis..

[34] Alton G.D., Berke O., Reid-Smith R., Ojkic D., Prescott J.F. (2009). Increase in seroprevalence of canine leptospirosis and its risk factors, Ontario 1998–2006. Can. J. Vet. Res..

[35] Lee H.S., Guptill L., Johnson A.J., Moore G.E. (2014). Signalment changes in canine leptospirosis between 1970 and 2009. J. Vet. Intern. Med..

[36] Geisen V., Stengel C., Brem S., Müller W., Greene C., Hartmann K. (2007). Canine leptospirosis infections—Clinical signs and outcome with different suspected *Leptospira* serogroups (42 cases). J. Small Anim. Pract..

[37] Adin C.A., Cowgill L.D. (2000). Treatment and outcome of dogs with leptospirosis: 36 cases (1990–1998). J. Am. Vet. Med. Assoc..

[38] Baker M.G., Lopez L.D., Cannon M.C., de Lisle G.W., Collins D.M. (2006). Continuing *Mycobacterium bovis* transmission from animals to humans in New Zealand. Epidemiol. Infect..

[39] Broughan J.M., Downs S.H., Crawshaw T.R., Upton P.A., Brewer J., Clifton-Hadley R.S. (2013). *Mycobacterium bovis* infections in domesticated non-bovine mammalian species. Part 1: Review of epidemiology and laboratory submissions in Great Britain 2004–2010. Vet. J..

[40] Gunn-Moore D.A., McFarland S.E., Brewer J.I., Crawshaw T.R., Clifton-Hadley R.S., Kovalik M., Shaw D.J. (2011). Mycobacterial disease in cats in Great Britain: I. Culture results, geographical distribution and clinical presentation of 339 cases. J. Feline Med. Surg..

[41] Erwin P.C., Bemis D.A., McCombs S.B., Sheeler L.L., Himelright I.M., Halford S.K., Diem L., Metchock B., Jones T.F., Schilling M.G. (2004). *Mycobacterium tuberculosis* transmission from human to canine. Emerg. Infect. Dis..

[42] Hackendahl N.C., Mawby D.I., Bemis D.A., Beazley S.L. (2004). Putative transmission of *Mycobacterium tuberculosis* infection from a human to a dog. J. Am. Vet. Med. Assoc..

[43] Posthaus H., Bodmer T., Alves L., Oevermann A., Schiller I., Rhodes S.G., Zimmerli S. (2011). Accidental infection of veterinary personnel with *Mycobacterium tuberculosis* at necropsy: A case study. Vet. Microbiol..

[44] Ner Z., Ross L.A., Horn M.V., Keens T.G., MacLaughlin E.F., Starnes V.A., Woo M.S. (2003). *Bordetella bronchiseptica* infection in pediatric lung transplant recipients. Pediatr. Transplant..

[45] Wernli D., Emonet S., Schrenzel J., Harbarth S. (2011). Evaluation of eight cases of confirmed Bordetella bronchiseptica infection and colonization over a 15-year period. Clin. Microbiol. Infect..

[46] Lenz J., Joffe D., Kauffman M., Zhang Y., LeJeune J. (2009). Perceptions, practices, and consequences associated with food-borne pathogens and the feeding of raw meat to dogs. Can. Vet. J..

[47] Wright J.G., Tengelsen L.A., Smith K.E., Bender J.B., Frank R.K., Grendon J.H., Rice D.H., Thiessen A.M., Gilbertson C.J., Sivapalasingam S. (2005). Multidrug-resistant *Salmonella Typhimurium* in four animal facilities. Emerg. Infect. Dis..

[48] Nicholson W.L., Paddock C.D., Demma L., Traeger M., Johnson B., Dickson J., McQuiston J., Swerdlow D. (2006). Rocky Mountain spotted fever in Arizona: Documentation of heavy environmental infestations of *Rhipicephalus sanguineus* at an endemic site. Ann. N. Y. Acad. Sci..

[49] Moro P., Schantz P.M. (2009). Echinococcosis: A review. Int. J. Infect. Dis..

[50] Deplazes P., van Knapen F., Schweiger A., Overgaauw P.A. (2011). Role of pet dogs and cats in the transmission of helminthic zoonoses in Europe, with a focus on echinococcosis and toxocarosis. Vet. Parasitol..

[51] Kern P., Ammon A., Kron M., Sinn G., Sander S., Petersen L.R., Gaus W., Kern P. (2004). Risk factors for alveolar echinococcosis in humans. Emerg. Infect. Dis..

[52] Kreidl P., Allerberger F., Judmaier G., Auer H., Aspöck H., Hall A.J. (1998). Domestic pets as risk factors for alveolar hydatid disease in Austria. Am. J. Epidemiol..

[53] Petavy A.F., Tenora F., Deblock S., Sergent V. (2000). *Echinococcus multilocularis* in domestic cats in France. A potential risk factor for alveolar hydatid disease contamination in humans. Vet. Parasitol..

[54] Otranto D., Sakru N., Testini G., Gürlü V.P., Yakar K., Lia R.P., Dantas-Torres F., Bain O. (2011). Case report: First evidence of human zoonotic infection by *Onchocerca lupi* (Spirurida, Onchocercidae). Am. J. Trop. Med. Hyg..

[55] Eberhard M.L., Ostovar G.A., Chundu K., Hobohm D., Feiz-Erfan I., Mathison B.A., Bishop H.S., Cantey P.T. (2013). Zoonotic *Onchocerca lupi* infection in a 22-month-old child in Arizona: First report in the United States and a review of the literature. Am. J. Trop. Med. Hyg..

[56] Petersen C.A. (2009). Leishmaniasis, an emerging disease found in companion animals in the United States. Top. Companion Anim Med..

[57] Rees R.K., Swartzberg J.E. (2011). Feline-transmitted sporotrichosis: A case study from California. Dermatol. Online J..

[58] Schubach A., Barros M.B., Wanke B. (2008). Epidemic sporotrichosis. Curr. Opin. Infect. Dis..

